# Osteogenic differentiation of adipose-derived canine mesenchymal stem cells seeded in porous calcium-phosphate scaffolds

**DOI:** 10.3389/fvets.2023.1149413

**Published:** 2023-06-02

**Authors:** David Herrera, Irene Lodoso-Torrecilla, Maria-Pau Ginebra, Katrin Rappe, Jordi Franch

**Affiliations:** ^1^Bone Regeneration Research Group, Department of Animal Medicine and Surgery, Veterinary Faculty, Autonomous University of Barcelona, Cerdanyola del Vallès, Spain; ^2^Biomaterials, Biomechanics and Tissue Engineering Group, Department of Materials Science and Engineering, Universitat Politècnica de Catalunya, Barcelona, Spain

**Keywords:** canine mesenchymal stem cell, bone graft substitute, β-tricalcium phosphate, CD90, ceramic scaffold, osteogenic differentiation

## Abstract

**Introduction:**

Engineered bone graft substitutes are a promising alternative and supplement to autologous bone grafts as treatments for bone healing impairment. Advances in human medicine extend an invitation to pursue these biomimetic strategies in animal patients, substantiated by the theory that specialized scaffolds, multipotent cells, and biological cues may be combined into a bioactive implant intended for the enhancement of tissue regeneration.

**Methods:**

This proof-of-concept study was designed to evaluate and validate the feasibility of beta-tricalcium phosphate foam scaffolds seeded with canine mesenchymal stem cells derived from adipose tissue. Cell-inoculated samples and sham controls were cultured statically for 72 hours in complete growth medium to evaluate seeding capacity, while a subset of loaded scaffolds was further induced with osteogenic culture medium for 21 days. Produced implants were characterized and validated with a combination of immunofluorescence and reflection confocal microscopy, scanning electron microscopy, and polymerase chain reaction to confirm osteogenic differentiation in tridimensional-induced samples.

**Results:**

After 72 hours of culture, all inoculated scaffolds presented widespread yet heterogeneous surface seeding, distinctively congregating stem cells around pore openings. Furthermore, at 21 days of osteogenic culture conditions, robust osteoblastic differentiation of the seeded cells was confirmed by the change of cell morphology and evident deposition of extra-cellular matrix, accompanied by mineralization and scaffold remodeling; furthermore, all induced cell-loaded implants lost specific stemness immunophenotype expression and simultaneously upregulated genomic expression of osteogenic genes Osterix and Ostecalcin.

**Conclusions:**

β-TCP bio-ceramic foam scaffolds proved to be suitable carriers and hosts of canine adipose-derived MSCs, promoting not only surface attachment and proliferation, but also demonstrating strong *in-vitro* osteogenic potential. Although this research provides satisfactory *in-vitro* validation for the conceptualization and feasibility of a canine bio-active bone implant, further testing such as patient safety, large-scale reproducibility, and quality assessment are needed for regulatory compliance in future commercial clinical applications.

## 1. Introduction

Recent bone-tissue engineering (BTE) tendencies depart from the basic concept of a bio-mimetic approach to regeneration and are sustained by the convergence of three main components: cells, scaffolds, and biological signaling (1, 2). In the field of orthopedic surgery, fresh autologous bone grafts remain the gold standard of bone healing enhancement because it combines osteoconduction and osteoinduction potentials, seemingly related to the high amount of stem cells, growth factors (GFs) and cancellous matrix present in bone marrow (3, 4). This treatment option is usually a first choice for bone augmentation in disabling and complicated conditions such as malunions, delayed unions, and nonunions, but also in other critical procedures such as arthrodesis; however, many concerns persist over the shortcomings associated with the procurement of these bone grafts, such as the morbidity and limited availability of graft material in donor sites (5). This last issue especially pertains to the veterinary medicine field, where a high percentage of patients are small sized and grafting is not only risky because of their thinner bone cortices, but also low in reward because the volume of available cancellous bone (graft material) is scarce and usually insufficient to address most conditions concerning bone healing impeachment.

In recent years BTE has gained popularity in research and development, fueled by unmet expectations and inherent limitations of the gold standard. Bone Grafts Substitutes (BGS) represent an auspicious alternative to the limitations of allografts and autografts (6, 7), although initial research has focused mostly on individual approaches instead of synergistic coalescence (8). A few examples of BGSs used and described in recent years are allogeneic or xenogeneic demineralized bone matrixes (DBMs), bioactive glass, and calcium phosphate ceramics, such as hydroxyapatite, tricalcium phosphate (TCP) and biphasic calcium phosphates (7). These last bio-ceramic materials perform as suitable BGSs because of their cell-homing capabilities and similarity in composition and ultrastructure to native cancellous bone (9), and are employed as scaffolds in the clinical setting on both human and animal patients. Lastly, fabrication methods including 3D printing, foaming and casting, among others, allow the calcium phosphates to be delivered in a platitude of architectural, surface and nanostructure configurations (10).

On the other hand, therapeutically delivered MSCs are known to immunomodulate and ameliorate local inflammation through various mechanisms (11, 12), also specializing and differentiating into osteoblasts, chondrocytes, fibroblasts, myocytes, and adipocytes as well (13, 14), thus potentially promoting both injury site homeostasis and augmenting tissular regeneration. Adult MSCs can be isolated from a plethora of tissues such as bone marrow, adipose tissue, muscle and periosteum (13) although many other tissues may be sources of multipotential MSCs (15). Consequently, modern cellular therapies require judicious selection of the source of stem cells based on previously characterized multipotentiality and immune-modulating privileges, but also for their *in-vitro* expansion rates, the availability of sourcing, and for their proven safe allogenic clinical use in canine patients (16–20). Furthermore, canine MSCs can be cryopreserved and banked (21, 22), with the potential to be revitalized and employed in experimental or clinical settings with consistency and availability.

Multiple studies have described the biology surrounding adipose-derived MSCs (23, 24), a type of adult stem cells readily available in the body and known for their excellent immunomodulatory properties (25, 26) and robust osteogenic (27, 28) and chondrogenic (29, 30) potentials. Consequently, adipose-derived MSCs represent a promising therapy for multiple diseases requiring immunomodulation and regeneration enhancement (31–33); a final consideration is that adipose-derived allogenic MSCs have proven to be safe in the veterinary clinical setting (34–36), nevertheless, literature reviews are important to contrast observations while evidence of higher quality is published regarding clinical efficacy for specific conditions (34–36).

In the case of bone healing, there is a multitude of factors involved in the specific osteogenic fate commitment of undifferentiated MSCs, the most important being mechanical factors, micromotion (strain), oxygen tension, local tridimensional configuration, and molecular signaling (37). Under the influence of these variants, differentiation of MSCs into osteoblasts goes through three sequential stages: first becoming an osteochondral progenitor cell, then an immature pre-osteoblast, and lastly, a mature osteoblast (38). It is important to note that cell therapies such as autologous and allogenic MSCs are rarely used as sole BGS, since these cells require specific growth development, tridimensional display (scaffolding, interconnection) and environmental cues (load, strain, surface charge) to be osteo-induced.

In short, it is proposed that the optimization of BGSs requires combining some of these materials and strategies into bioactive bone implants. Stem cell or gene therapies, along with cytotactic signaling, GFs, osteogenic enhancement treatments such as bone morphogenic proteins (BMPs), and surface treatments may be merged interchangeably for improved material-cell interactions and seeming less *in-vivo* osteointegration of the implant (39). When merged, these synthetic and biological products also known as orthobiologics, offer the opportunity to customize bioactive implants and organoids designed to attend specific clinical and anatomical situations with tailored potentials (1). Today, as BTE grows in refinement and standardization, so does the demand for clinical application: in human medicine its market (i.e., ortho biologic*s*) was size capped at USD 5 billion in 2017 (40), roughly 10% of the total orthopedic industry market size. Although there is no data for the market size of veterinary ortho biologics, the overall orthopedic pet market size was capped at USD 530 million in 2021 and expanding exponentially (41), and this emerging segment will likely follow an exponential growth as technologies are refined and quality evidence is produced.

The following is an *in-vitro* proof of concept (POC) and methods study involving a biomimetic bone implant, an evidence-based (2, 42, 43) BGS inspired by naturally occurring mechanisms of bone repair. The clinical relevance of these bioactive implants lies in their potential as possible therapy for mechanically unloaded segmental bone defects or critical defects, late unions, atrophic non-unions and arthrodesis in canine patients. We hypothesize that the architecture, microstructure, and surface configuration of surface-treated calcium phosphate implants will permit *in-vitro* canine MSC adhesion and transport. Furthermore, we estimate that differentiation of the scaffold-attached cells into pre-osteoblasts and osteoblasts could also be feasible under certain media-inducing conditions, thus enhancing its osteoinductive, osteoconductive, and osteogenic potentials before theoretical clinical implantation.

## 2. Materials and methods

### 2.1. Experimental design summary and justification

**Figure 1:**
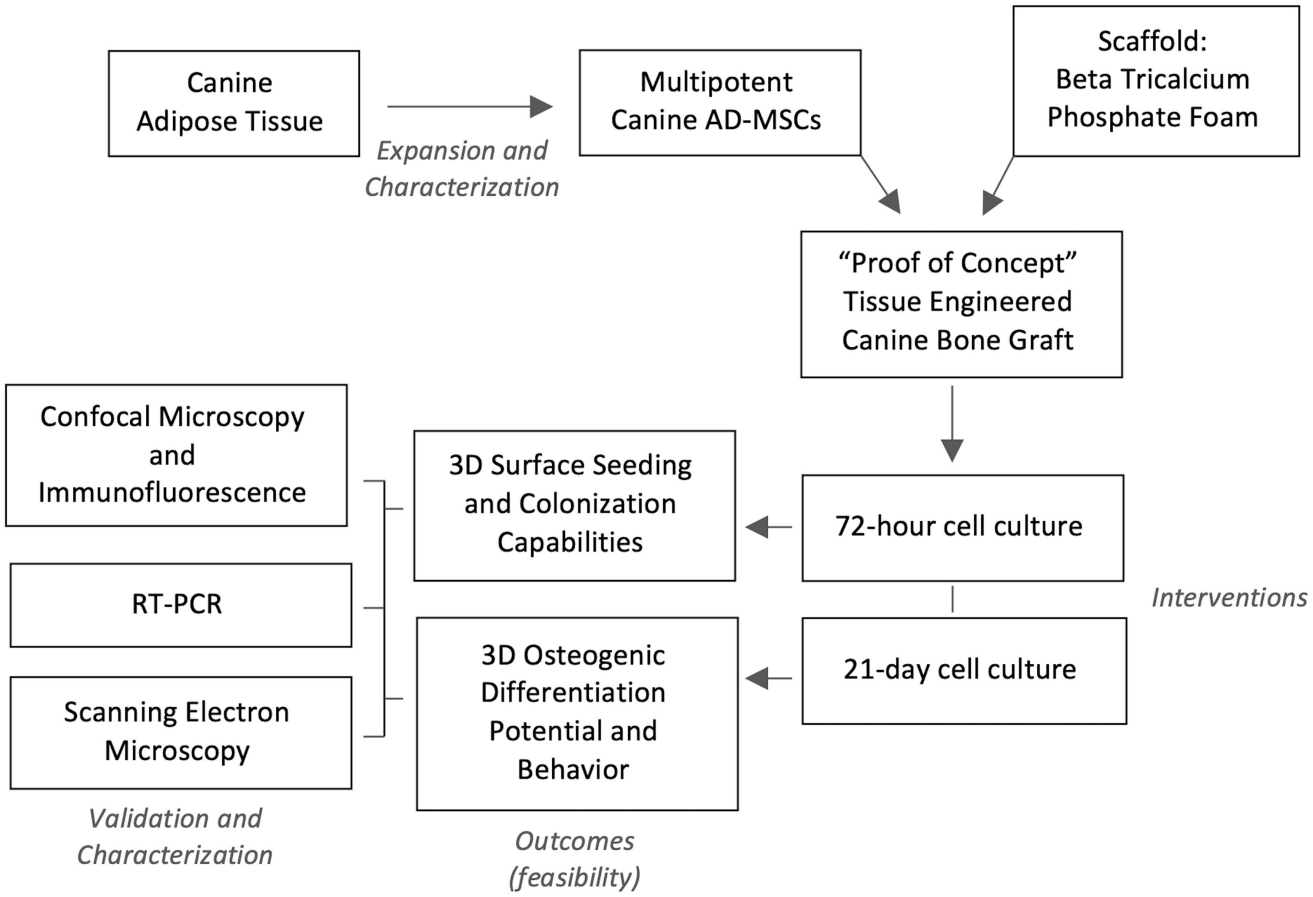
Experimental design flow. Four lines of characterized canine adipose-derived mesenchymal stem cells (AD-MSC) were expanded and seeded in Beta Tricalcium Phosphate (β-TCP) scaffolds for seeding and colonization evaluation at 72 h, while a seeded sample subset was differentiated osteogenically for 21 days. Validation of confected bioactive scaffolds was performed by confocal microscopy with immunofluorescence, sample surface scanning electron microscopy and osteogenic genetic profiling through reverse transcription polymerase chain reaction (RT-PCR).

A conceptualized experimental design flow chart is depicted in Figure 1. In short, a batch production of similar 3D foam ceramic implants was inoculated with four different canine donor lines of MSCs, these seeded implants were then evaluated for their capability to promote *in-vitro* cell adhesion and osteogenic differentiation. A subset of sham scaffolds (i.e., not seeded with cells) was cultured and induced in the same conditions as a control.

In this study, β-TCP foams were selected as cell carriers for undifferentiated MSCs because of the materials reported biocompatibility, safety and efficacy on canine models (44) and because of its proven ectopic osteoconduction and osteoinduction capabilities (45), when configured with an architecture of porous interconnections and spherical concave surfaces. The final material selection and configuration were based on the aforementioned observations and considering biomechanical properties published in other human and canine models (46, 47).

### 2.2. Confection of β-tricalcium phosphate 3D foams (cell carriers)

Rigid ceramic scaffolds with porous surfaces and interconnection were obtained employing a foaming technique, which conferred a unique macrostructure to the construct consequence of the random allocation of air bubbles created during the mixing process. The setting and heat treatment of the mineral material eventually yielded a scaffold with a high number of concave pores with various sizes and interconnected paths.

Scaffolds were prepared similarly to a previous study that linked ceramic scaffold architecture and osteoinduction capabilities in a canine ectopic implantation model (45). In short, a soft paste was obtained by blending a solid phase of 98% α-TCP and 2% precipitated hydroxyapatite (PHA, Merck KGaA; Darmstadt, Germany) with an aqueous solution of 1% polysorbate 80 (Tween 80, Sigma-Aldrich; St. Louis, MO, United States), at a liquid to powder ratio of 0.65 mL/g. Foaming of the mix was carried out with a domestic food mixer for 30 s at 7,000 rpm and allowed for a homogeneous blend while also introducing variably sized air bubbles throughout the complete volume of the mixture.

Next, the foamed liquid paste was then poured on Teflon cylindrical molds with a diameter of 5 mm and a height of 5 mm. These filled molds were then placed in a laboratory-heated water bath for 90 m to promote mineral binding, following immersion in deionized at 37° C for a total of 7d to allow for the hydrolysis reaction of α-TCP to calcium deficient hydroxyapatite (CDHA) to take place, according to the following reaction (Equation 1):


(1) $$\mathrm{3Ca3}\left(PO4\right)2+H2O\to Ca9\left(PO4\right)5\left(HPO4\right)OH$$

After hydrolysis, a final heat treatment was applied to all implants in a furnace (CRN-58-1, Hobersal; Barcelona, Spain) at 1,100°C for 15 h, to allow for the conversion of CDHA into β-TCP; this process preserves the overall architecture and macrostructure, but alters the material’s surface at a microscopic level, shifting from protruding mineral spicules (i.e., whiskers) typical of CDHA to an even, smooth bedrock-like surface, characteristic of β-TCP. The resulting scaffolds presented a mean total porosity and macro porosity of *ca.* 65 and 50%, respectively, with a mean macropore size of 232 μm and a specific surface area (SSA) of 0.46 m^2^/g.

Lastly, each scaffold was coated with poly-D-lysine (PDL) (Poly-D-Lysine; Gibco; Massachusetts, United States) to improve the electrostatic interaction between cells and scaffold surface. Briefly, a total of 0.3 mL of PDL at a concentration of 50 μg/mL was poured gently and covered all surfaces of the implant with the aid of a micropipette, under aseptic conditions in a laminar flow hood. This process was repeated until the whole implant surface was sufficiently covered. Excess PDL was gravity drained in a slanted dish for 5 min, followed by a final rinse with sterile Milli-Q water. Coated scaffolds were then dried for 3 h at room temperature (RT) in sterile containers; lastly, samples were aseptically sealed and stored at 4°C overnight for experimental use.

### 2.3. Canine adipose-derived mesenchymal stem cells

In this study, cryopreserved adipose-derived MSCs procured from canine falciform ligaments were obtained from the Animal Stem Cell Bank at the UAB Research Park (Autonomous University of Barcelona; Bellaterra, Spain). Characterization of these canine MSCs was performed previous to experimentation through the confirmation of plastic adherence, fibroblast-like morphology, proliferating capabilities and immuno-phenotypic expression and absence of canine-specific antibodies (−CD34, +CD44, −CD45, +CD90). Furthermore, the determination of RNA expression of multipotent associated genes OCT4 and NANOG along with two-dimensional tri-linear *in-vitro* differentiation was performed during banking as compiling evidence of cellular identity and similarly to recently proposed minimal criteria for reporting veterinary and animal medicine research for mesenchymal stromal/stem cells in orthopedic applications (48).

When prepped to be revitalized, frozen vials containing passage-1 cells were thawed and seeded in plastic flasks with an initial cell density of 1 × 10^4^ cells per cm^2^ in complete growth medium (CGM), composed of low glucose Dulbecco Modified Eagle’s Medium (ThermoFisher DMEM; Massachusetts, United States) supplemented with 2 nM of L-glutamine (Gibco L-Glutamine 200 mM; Massachusetts, United States) and 10% v/v fetal bovine serum (FBS) (Gibco Fetal Bovine Serum, EU qualified; Brazil, Lot 2010343S2); and incubated at 37°C, 5% CO2, and 95% humidity until a confluence of 80% for experimental use.

### 2.4. Implant surface cell seeding

Once scaffolds were pre-coated and dry, the seeding protocol was initiated. For this, a cellular inoculum was confected by suspending 1 × 10^6^, passage-2 (P2) canine MSCs in 1 mL of CGM. With a micropipette and a sterile tip, the cell-rich suspension was applied systematically, visually confirming the permeability of the implant that occurs once the superficial tension of drops has been broken. Finally, seeded implants were then placed in the incubator at 37°C, 5% CO_2_, and 95% humidity and left to adhere for at least 1 h. After that, the process was repeated by carefully aspirating the remanent supernatant and flipping the scaffold with fine-point forceps, and reapplying the inoculation on the remaining unseeded surfaces. Once covered evenly, carrier implants were placed in a vertical position in 12-well plates for maintenance and interventions.

### 2.5. Study groups

Quintuplicates of revitalized canine adipose-derived MSCs originating from four different individuals were allocated intendedly into two study groups in accordance to their fate: Set 1 (S1, *n* = 10) of samples was incubated statically in CGM for 72 h, without medium repletion in the time given, to assess the feasibility of the cell seeding technique and short-term proliferation potential of MSCs in the tri-dimensional configuration the scaffold’s topography. Similarly, Set 2 (S2, *n* = 10) of seeded implants was left to adhere in CGM for 72 h but instead introduced to an osteogenic induction medium (OIM) (StemPro Osteogenesis Differentiation Kit, Gibco; Massachusetts, United States; complemented with 10% FBS) for an extended total of 21 days, with OIM exchanges performed every 3 or 4 days to promote nutrient availability. This 3-week osteoinduction essay is intended to demonstrate the feasibility of canine *in-vitro* osteogenic differentiation in a tri-dimensional calcium phosphate construct. Lastly, a subset of sham control pre-coated scaffolds with no cells seeded (*n* = 4) was also cultured synchronically and stained in the same conditions as both study groups S1 and S2, in order to evidence any cell-associated scaffold degradation or remodeling.

### 2.6. Outcome measurement and implant analysis

#### 2.6.1. Immunocytochemistry and confocal microscopy

When up for analysis, the culture medium was aspirated and discarded. The process was initiated with the permeabilization of the attached cells, performed first by fixating implants in 4% paraformaldehyde at 1 mL/implant for 10 m at RT. Next, the fixation solution was aspirated, and each scaffold was thoroughly washed twice with cold phosphate-buffered saline (PBS), which followed cell permeabilization and priming, achieved by incubating implants with 0.5% Triton X-100 in PBS (PBST) at RT for 10 m. Next, PBST was aspirated and attached cells were washed with PBS Tween (0.1% Tween 20 in PBS), three times for 5 m. Following this, unspecific binding sites were inhibited by exposing cells to a blocking buffer (1% bovine serum albumin in PBST) for 1 h at RT.

The cell-surface glycoproteins CD90 and CD44 are reportedly expressed and associated with the cellular state of non-differentiation in MSCs (49) and are currently used for immunophenotype profiling of canine MSC stemness (50). In this study, a canine-specific anti-CD90 antibody (eBioscience Anti-Dog CD90 (Thy-1) monoclonal anti-body; LOT 2252668. ThermoFisher, Massachusetts, United States) conjugated with a phycoerythrin (PE) fluorophore, was employed on all samples at a dilution of 1:50, according to manufacturer’s indications and internal pre-verification. Finally, samples were left to conjugate in light-absent conditions at 4°C for at least 4 h. All implants were repeatedly washed with cold PBS before secondary staining to remove excess unbound antibodies.

In order to achieve a contrasted image of the attached MSCs, the nucleus DNA was counter-stained with 4′,6-diamidino-2-phenylindole (DAPI) (Invitrogen NucBlue Fixed Cell Ready Probes Reagent; Oregon, United States) according to manufacturer’s instructions. Before optical fluorescent microscopy analysis, one drop of reagent was applied on each side of each implant and left to incubate in dark conditions at RT for 5 m. Surface analysis and detection of fluorescent markers in our set of samples were possible utilizing a confocal laser scanning microscope Leica SP5 (Leica Microsystems CMS GmbH; Mannheim, Germany), detecting the signal for CD90 (excitation 561 nm, detection 570 mm-685 nm; showed in green), the nuclei stained with DAPI blue (excitation 405 nm, detection 415 mm–520 nm; showed in blue) and the light reflected on the scaffold’s surface (excitation 561 nm, detection 555 mm-567 nm). Tridimensional volume rendering and additional image processing were carried out with Imaris visualization software (Bitmap Imaris RRID:SCR_007370, http://www.bitplane.com/imaris/imaris; Belfast, UK).

#### 2.6.2. Ultrastructural analysis

Scanning Electron Microscopy (SEM) was performed to obtain a microscopic qualitative assessment of loaded implants and controls via scanning after sample processing. Cell-implanted scaffolds from each cell line were fixed in 2.5% glutaraldehyde diluted in cacodylate buffer (CB) 0.1 M for 2 h. Next, samples were rinsed with CB and left to dry at RT, followed by post-fixation, performed by exposing samples to vapors of 1% osmium tetroxide containing 0.8% ferrocyanide for 2 h and dehydrated in increasing concentrations of ethanol (50, 70, 90, 96, 100%). Finally, samples were chemically dried with hexamethyldisilazane (HMDS) and coated thoroughly with Au/Pd. Seeded and mock scaffolds were evaluated with a scanning electron microscope Zeiss Evo MA10 (Carl Zeiss Microscopy GmbH; Oberkochen, GER), proprietary SmartSEM image acquisition software was used to scan and record implant surface and to evaluate the material composition of observed ECM.

#### 2.6.3. Digital nucleus analysis count

A novel digital approach to surface cell-seeding density estimation in 3D environments was proposed for this study by employing the images obtained from confocal immunocytochemistry in a specific fluorescent range, intending to create a nucleus-only image window of sequential microphotographs.

**Figure 2:**
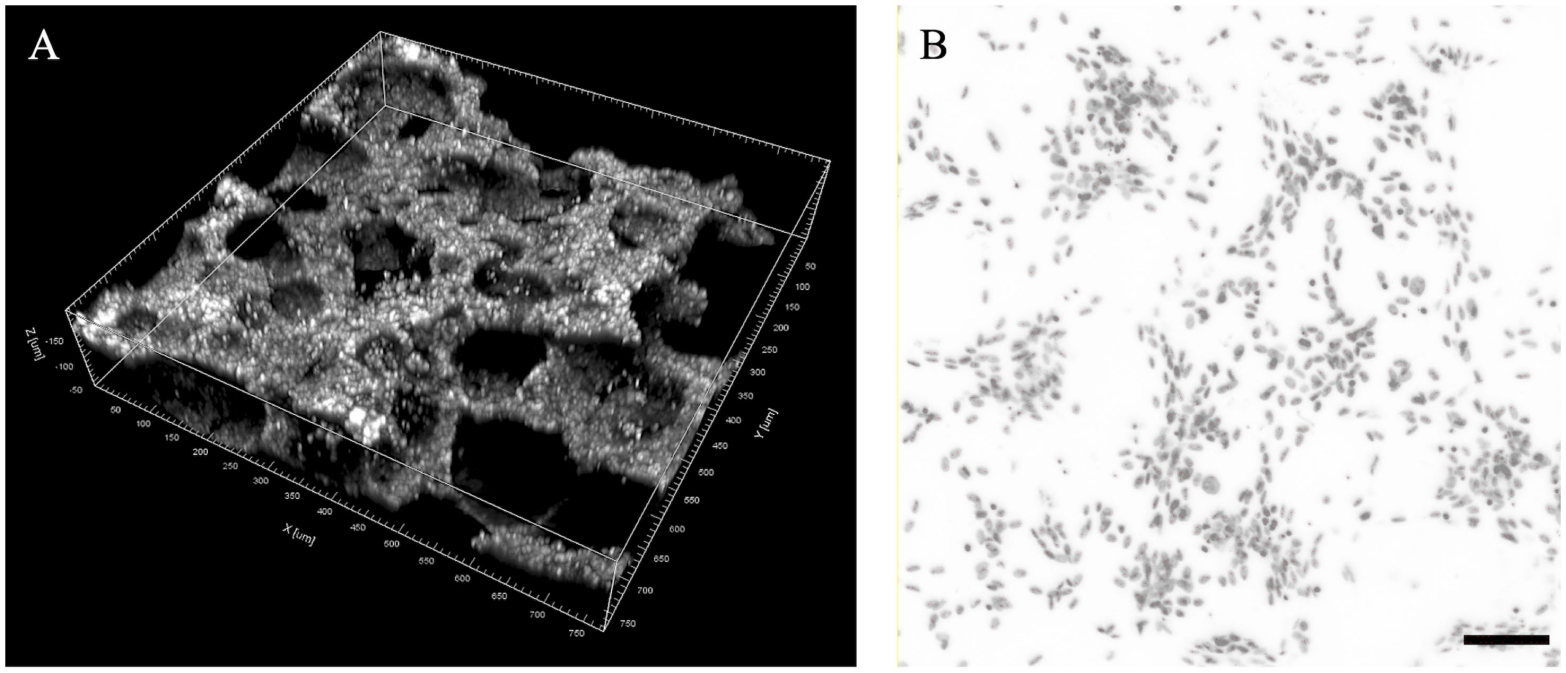
Proposed regions of interest (ROIs) for the analysis of cell seeding density in foamed calcium phosphate cylinders. **(A)** ROI volume dimensions (750 × 750 × 150 μm) in a tridimensional digital reconstruction of a region example; **(B)** Example of digital threshold analysis of DAPI stained nucleus for cell seeding density estimation (scale bar: 100  μm).

Considering the porous and irregular topography of the phosphate scaffolds, six regions of interest (ROIs) with symmetrical distribution were defined to obtain tridimensional digital volumes (750 μm × 750 μm × 150 μm) of the implant’s surface (Figure 2). This was achieved using confocal microscopy, specifically by choosing the DAPI stained window, detected at 415–520 nm (observed as blue nuclei over a black background). This image allowed for a process of automated digital counting of single-color images, converting blue color to greyscale and thus creating a binary image of particles (black = 0 and white = 255 scale). Next, the standard parameters for threshold tolerance such as size (pixels) and shape (circularity) were set to define the instances in which the grey nuclei were counted. Based on this post-production digital analysis, the total number of viable cells was determined with an Image-based Tool for Counting Nuclei (ITCN). The number of viable cells on each one of the ROIs was considered the average of 5 replicas, and the total cell-surface density for each bioactive implant reported as a total of viable cells per cubic μm (cells/μm^3^).

#### 2.6.4. Reverse transcription polymerase chain reaction

Determination of RNA expression of multipotent associated genes Osteocalcin (OC) and Osterix (OSX) was performed similarly to a previously published study (28) on all S2 implants (21d differentiation induction) with the intention to genetically confirm the identity shift from multipotent MSC to pre-osteoblast and osteoblast (osteogenic differentiation). Gene sequences used in this study are summarized in Table 1.

**Table 1:** Gene primers used in rt-PCR for the evaluation of osteogenic differentiation in canine MSCs.

Gene	Forward	Reverse
*OC*	*GAGGGCAGCGAGGTGGTGAG*	*TCAGCCAGCTCGTCACAGTTGG*
*OSX*	*ACGACACTGGGCAAAGCAG*	*CATGTCCAGGGAGGTGTAGAC*
*GAPDH* *	*GGAGAAAGCTGCCAAATATG*	*ACCAGGAAATGAGCTTGACA*

* Housekeeping gene, OC, Osteocalcin; OSX, Osterix.

In order to process the bio-active scaffolds, liquid nitrogen gases were employed to dry-freeze samples and facilitate homogeneous mechanical breakdown in a ceramic mortar. Shattered fragments were then processed with a standard mRNA extraction kit (Qiagen RNeasy Mini Kit; Dusseldorf, GER), using GAPDH as a housekeeping gene for reference confirmation of upregulated expression.

### 2.7. Statistical and digital image analysis

Analysis of variables associated with nucleus count and distribution was performed through basic descriptive statistics [mean and standard deviation (SD)]. Due to the experimental and confirmatory nature of this study, the limited sample power and its descriptive statistical analysis, inter-sampler variability and statistical significance of observed differences were not pursued and considered beyond the objective of this study.

An open-source image processing package was employed for scaling, editing, and bioimage processing (ImageJ, image processing package; https://imagej.nih.gov/ij/, U.S. National Institutes of Health, Bethesda, Maryland, United States): (51), and open-source ITCN plug-in for the binary image particle counting (UCSB Center for Bio-Image Informatics, https://bioimage.ucsb.edu/sites/bioimage.ucsb.edu/files/docs/ictn_.tar; California, United States).

## 3. Results

### 3.1. Scaffolds as carriers of multipotential stem cells

#### 3.1.1. Microscopic evaluation of scaffolds after 72  h of culture

**Figure 3:**
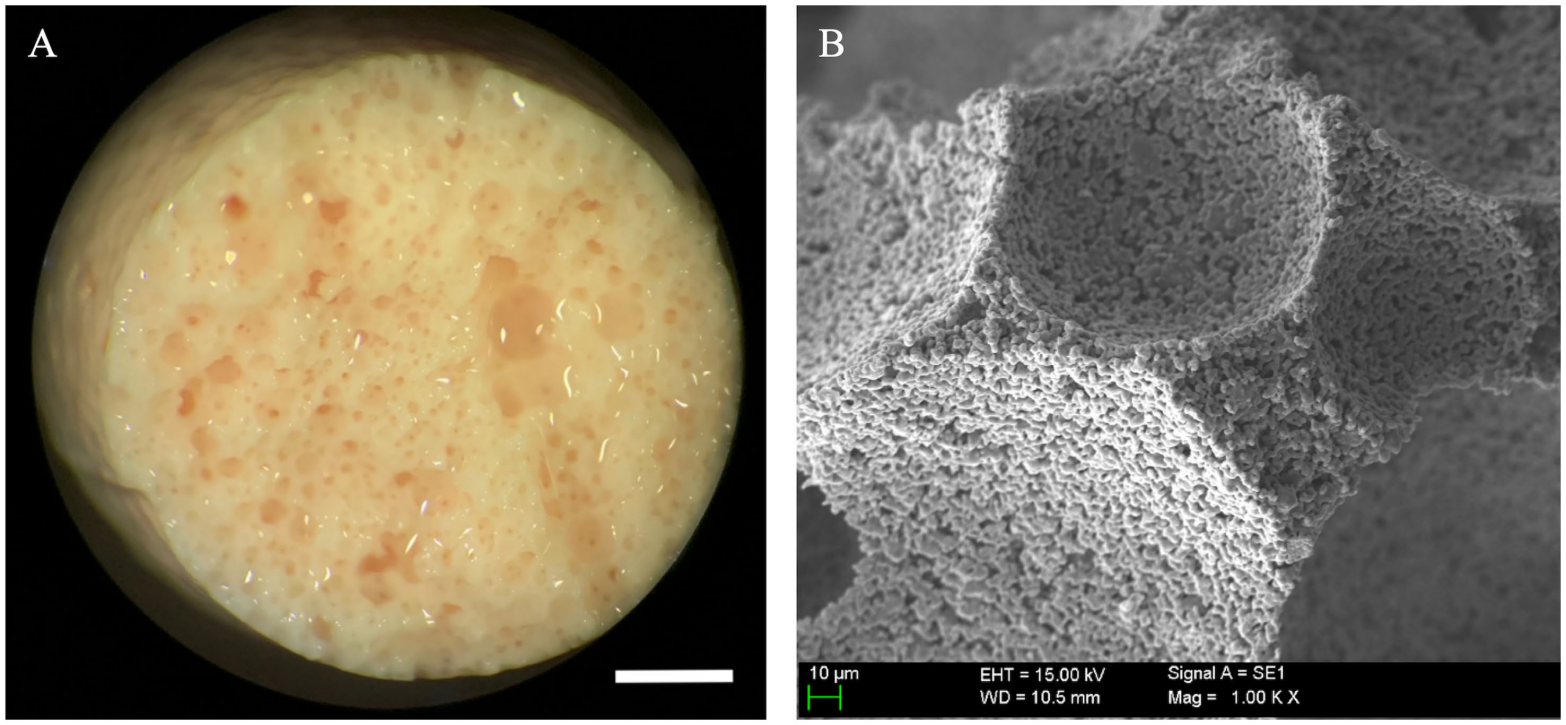
Macroscopic and ultrastructural aspect of unloaded scaffolds. **(A)** Beta tricalcium phosphate scaffold (β-TCP) foams maintained structural integrity after a 21-day sham culture with no cells seeded; these were employed as controls to assess gross and microscopic changes associated with mesenchymal stem cell (MSC) seeding and specialization (scale bar: 1 mm). **(B)** β-TCP foam at ultra-high magnification, note the preserved empty scaffold concavities and smooth, granular surface configuration typical of β-TCP (scale bar: 10 μm).

Scaffolds were observed first with an inverted light microscope to completely evaluate all the implant’s surface, with no evident signs of structural degradation or gross contamination. In Figure 3 a sham scaffold sample is shown and used to compare and detect macro and microscopic changes that may be associated with cell seeding and colonization.

**Figure 4:**
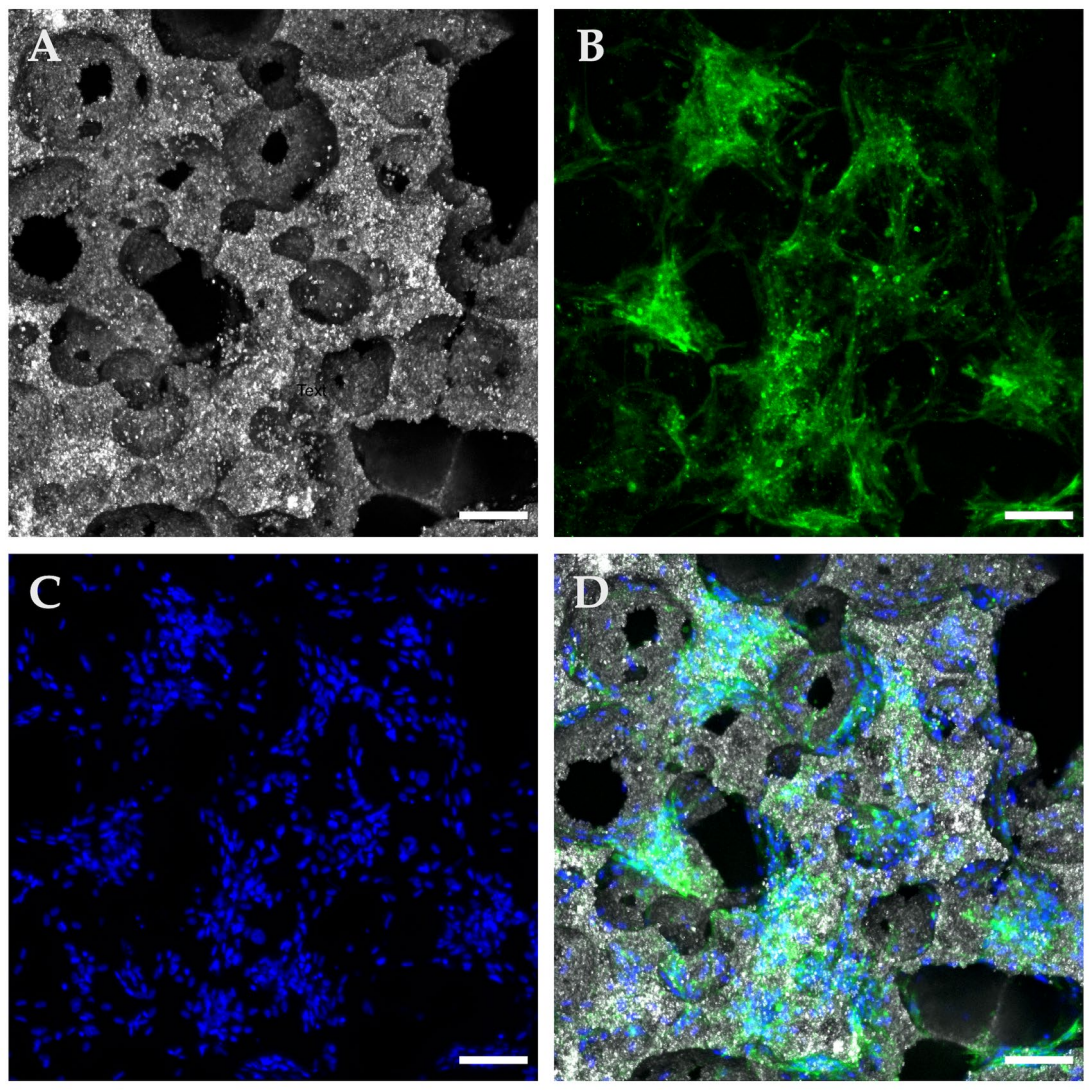
Confocal microscopy with immunofluorescence of a “Bottom” ROI in a seeded β-TCP scaffold after 72 h of culture in complete growth medium. **(A)** Confocal microscopy in reflection mode allows for a concise depiction of the scaffold surface and pore architecture. **(B)** Immunofluorescent anti-CD90 antibodies confirm multipotential identity and at the same time delimit the cytoplasmic surface. **(C)** DAPI blue nucleus staining allows for cells counting and distributions analysis. **(D)** Composite image of the panels, showing widespread coverage of the implant’s surface with CD90 positive cells (scale bar: 100 μm).

**Figure 5:**
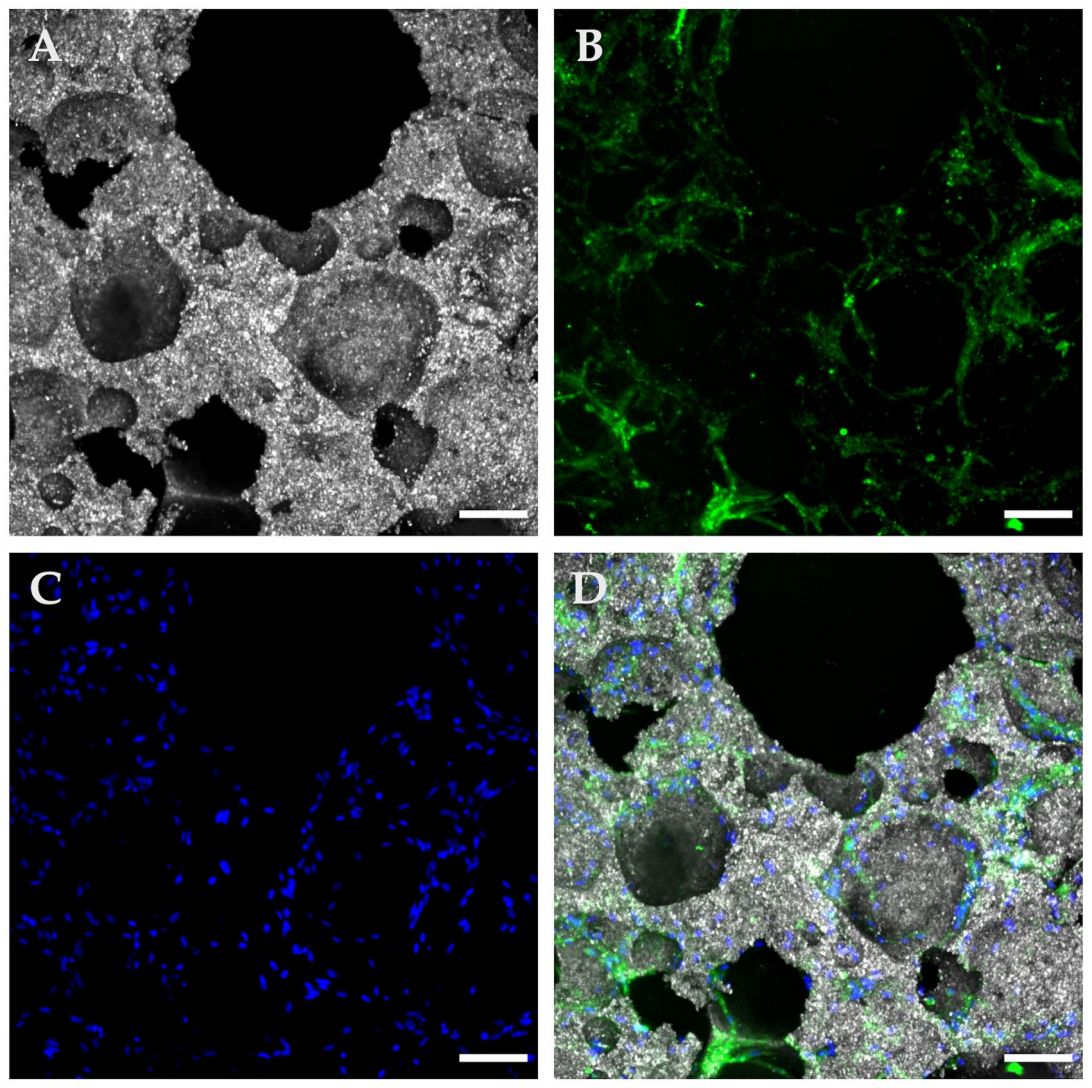
Confocal microscopy with immunofluorescence of a “Top” ROI in a seeded β-TCP scaffold after 72 hours of culture in a complete growth medium. **(A)** The size variance and interconnections of pore openings are evident in this reflection mode of the scaffold’s topography; **(B)** A fluorescent green light signal corresponding to anti-CD90 antibodies allows confirmation of suspected immunophenotype and at the same time, a clear depiction of the cytoplasmic configuration of the attached cells. **(C)** DAPI blue nucleus staining contributed to the assessment of cell viability, population, and distribution; **(D)** A composite image of the panels A, B, and D employed as an integrative tool to quantitatively and qualitatively characterize the seeded implant (scale bar: 100 μm).

S1 samples observed in confocal microscopy consistently confirmed ample cytoplasmic expression of CD90, seen as a bright green signal. Correspondingly, attached cells were positive for DAPI blue, which confirmed viability and adhesion. In Figure 4, a confocal microscopy image of a bottom region shows copious amounts of viable canine MSCs attached in various locations of a loaded scaffold after 72 h of culture, predominantly congregating alongside pore contours and openings. Conversely, a positive but weaker cell seeding density was visually appreciated in top regions, as detailed in Figure 5 where attached, viable CD90 positive cells colonized the scaffold’s surfaces, overlapping each other at some points and favoriting establishment at pore contours and flat surfaces.

#### 3.1.2. Digital nucleus count

Cell-seeding density averages and overall regional means were calculated for all ROIs and samples; both summarized in Table 2. The overall sample average of cell surface seeding density achieved after a 72 h static cell culture was 556.8 viable cells/μm^2^ (SD 100.9). An average of 1039.4 cells/μm^2^ (SD 123) were detected on “Bottom” ROIs, while “Lateral” ROIs displayed a mean of 127.1 with an SD of 37.6. Finally, “Top” ROIs were successfully seeded with an average of 504 cells/μm^2^ (SD 203.6). There were no empty ROIs at the time of analysis, and no signal was detected for DAPI blue on sham controls thus nucleus count remained cero.

**Table 2:** Observed cell density in ROIs obtained via digitalized nucleus count.

ROI	Sample average (cells/μm^2^)				Region average (cells/μm^2^)	SD
	1	2	3	4		
Top	397	313	779.5	526.5	504	203.6
Laterals	105	131	178.5	94	127.1	37.6
Bottom	915.5	1137.5	1152.5	952	1039.4	123

ROI: region of interest; SD: standard deviation.

#### 3.1.3. Ultrastructural evaluation of surface seeding

**Figure 6:**
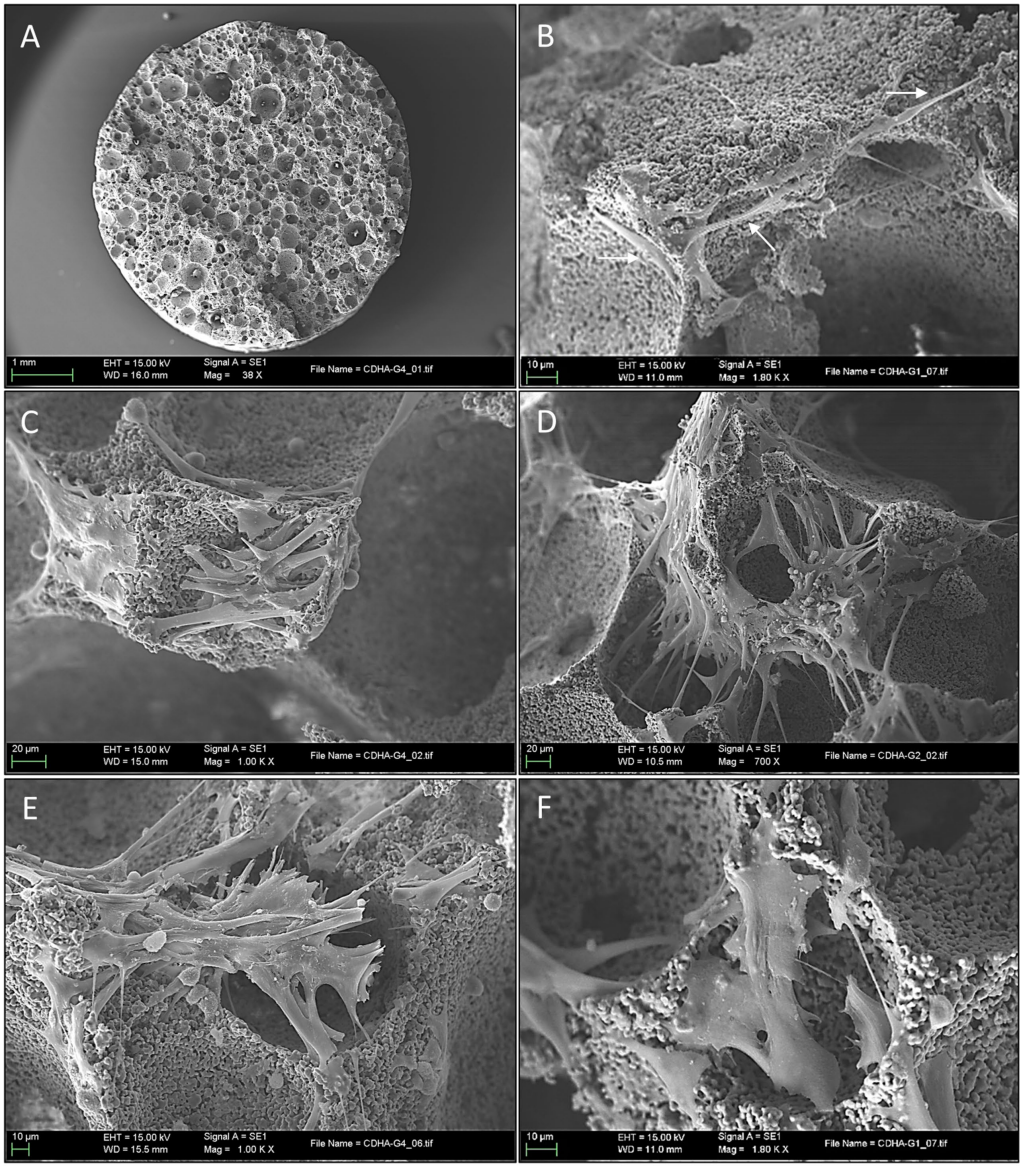
Scanning electron microscopy images of mesenchymal stem cells attachment and proliferation in scaffold surfaces. **(A)** Low magnification appearance of the scaffold 3 days after seeding, the porous structure is clearly defined similarly to mock samples (scale bar: 1 mm); **(B)** Scanning electron microscopy (SEM) at high magnification 3 days after seeding, note the presence of the spindle cells placed in the pores’ borders (arrows) (scale bar: 10 μm); **(C)** SEM high magnification appearance of a shallow pore 3 days after seeding. Note the rapid progression of the stem cell invasion that rapidly develops larger cytoplasmic prolongations covering the pore’s surface (scale bar: 20 μm); **(D)** Here, MSCs are seeing extending and projecting thin pseudopodia creating “bridging connections” between the pore’s margins (scale bar: 20 μm); **(E)** A progressive flattening of the stem cells loosening its thin spindle aspect and the presence of short and thick cytoplasmic prolongations across the pore opening (scale bar: 10 μm); **(F)** Widening stance of in an attempt to cover the pore opening (scale bar: 10 μm).

After 3 days of culture, the macroscopic appearance of S1 samples did not differ significantly from that of the unseeded control scaffolds (Figure 6A). However, at high magnifications, the SEM images showed the presence and attachment of multiple cells to the scaffolds’ pores and contours (Figure 6B); in certain areas, the entire pore margins were completely lined with MSCs. Cell progression and behavior patterns seemed to depend on the size of the pore they were placed on: in the case of shallow pores, the cells rapidly invaded the bottom of the pore from the perimetric borders and once there, started to quickly develop larger filopodia and lamellipodia that covered almost completely the bottom surface of the pores (Figure 6C), progressively invading down the walls until reaching their bottom to congregate with other cells. This alternative approach of cell distributions was infrequent but quite efficient to address shallow or flattened pores and was accompanied also by a change of their stance and shape once their final location was decided.

In contrast, most of the deeper pores were approached with cells emitting longer cytoplasmic extensions towards the opposite edges of the circumference, thus creating “bridging connections” (Figure 6D). Pore-covering cells began to modify their morphology, adapting a more flattened and wider shape with a considerably diminishing filopodia length and volume (thus implying reduced cell mobility) (Figure 6E). Cytoplasmatic changes in shape also conferred a more flattened appearance, which allowed an efficient covering of the pore opening by the progressive layering of multiple cells and lamellipodia (Figure 6F).

### 3.2. Osteogenic β-TCP scaffolds

#### 3.2.1. Confocal microscopy analysis at 21  days of culture

**Figure 7:**
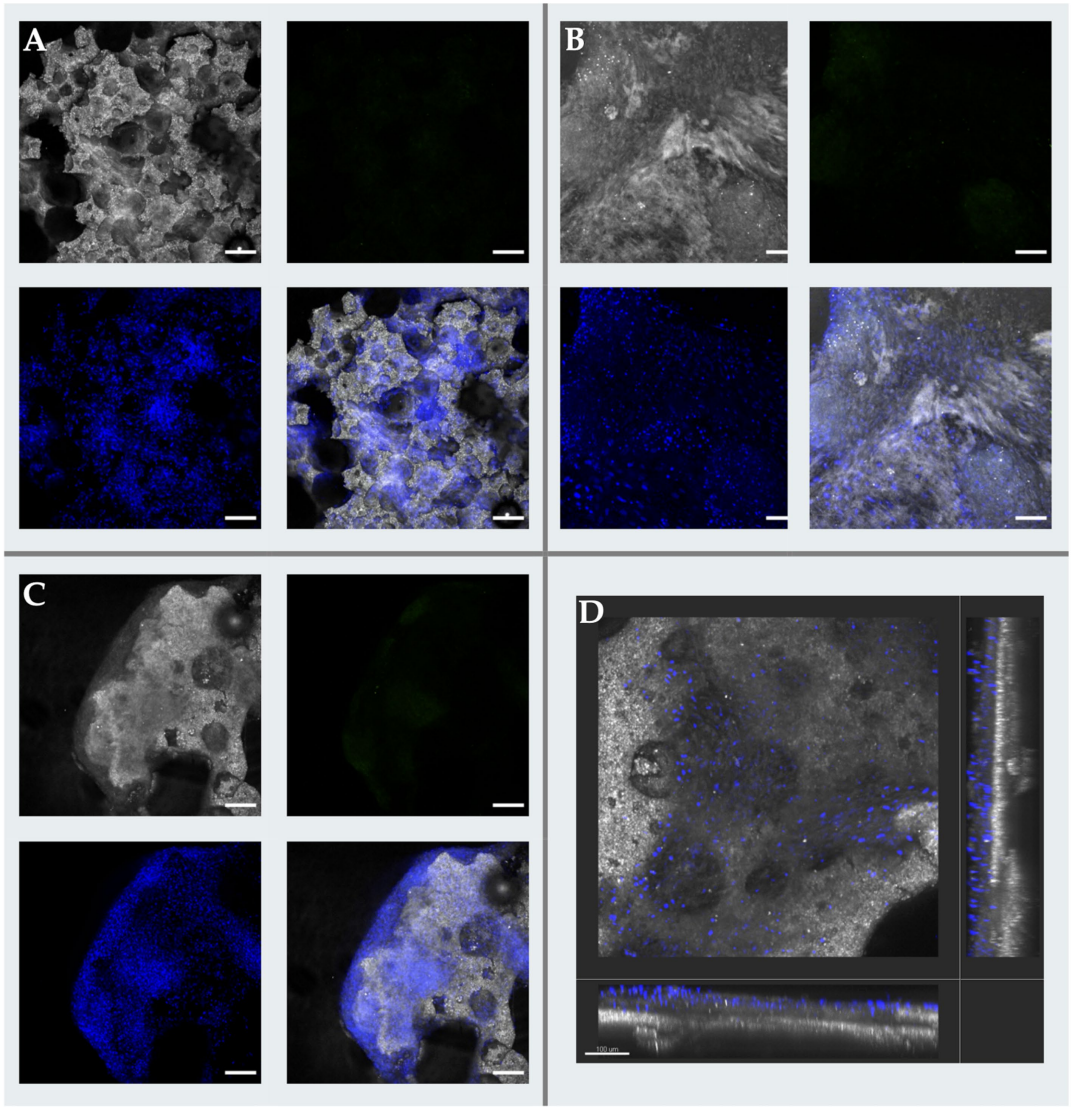
Confocal microscopy micrographs of osteogenic scaffolds. **(A)** Image depicting a “Top” Region of Interest (ROI) with extensive cell coverage characterized by discrete extra cellular matrix (ECM) deposition across de pore edges and over the scaffolds surface (scale bar: 100 μm); **(B)** “Bottom” ROI presenting gross and extensive ECM buildup over the implant accompanied with high cellularity (scale bar: 100 μm); **(C)** Transposition of the MSCs from the scaffold’s to the ECM was consistently observed throughout samples, associated to cell embedding in newly formed tissue and due to advanced osteogenic differentiation (scale bar: 200 μm); **(D)** Tridimensional reconstruction a surface analysis of a scaffold segment in which pores are covered with cells remaining on the upper layering of the recently created ECM (scale bar: 100 μm).

When analyzed using immunofluorescence, osteogenically differentiated samples (S2) emitted trace or absent CD90 PE signal, suggestive of a shift from cell multipotency to specialization. Strong DAPI signals confirmed cell viability and an increased number of attached cells when compared to S1 group. On non-fluorescent confocal imaging, samples also exhibited a thick layer of material deposition, presumably partially mineralized ECM by the intensity of confocal reflection, which seemed to cover ample surface areas while containing a considerable amount of cell nuclei embedded within. A pictogram of immunofluorescence for all sample lines and groups qualitatively summarizes observed results in Figure 7.

#### 3.2.2. Ultrastructural evaluation of osteogenic scaffolds

**Figure 8:**
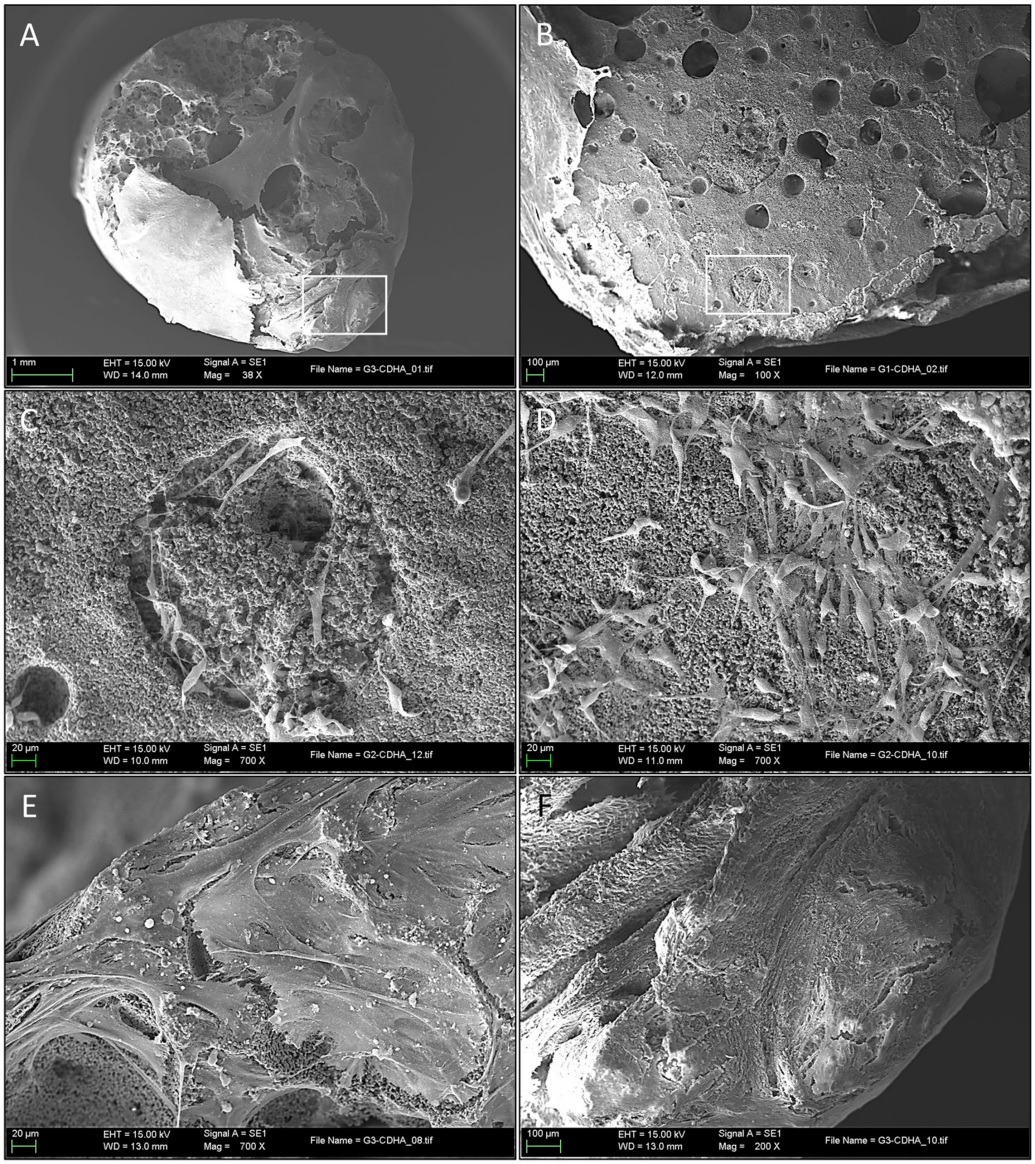
Ultrastructural evaluation of mineralized osteogenic scaffolds after 21 days of culture. **(A)** Note how the porous scaffold structure is mostly covered by a thick mineralized tissular mantle (scale bar: 1 mm); **(B)** A partially calcified tissue, composed of extracellular matrix (ECM) is covering the scaffold’s surface (left side of the picture). Mineral deposition and pore occlusion were no seen homogenously throughout the surface, robust new bone formation in some parts can be contrasted with other areas in which pores remain empty (scale bar: 100 µm); **(C)** Scanning electron microscopy (SEM) of a pore at 21 days after seeding. Here, differentiated cells (osteoblasts) are seen congregated around a pore; image inlet from Figure 8B (scale bar: 20 µm); **(D)** SEM high magnification appearance of the scaffold’s surface 21 days after seeding depicting a high concentration of cells (osteoblasts) progressing in a non-porous (flat) area of the scaffold surface, note de round and short morphology of differentiated cells (scale bar: 20 µm); **(E)** Both porous and non-porous areas of the scaffold surface are progressively covered by grossly detectable calcified ECM (scale bar: 20 µm); **(F)** Formation of thick calcified tissue in this area impedes the visualization of the porous surface of the scaffold and is associated with a mature state of tissue calcification; image inlet from Figure 8A (scale bar: 100 µm).

At 21 days of osteogenic culture, all the S2 samples showed a uniform macroscopic appearance. At low magnifications, the high porosity appearance of the scaffold observed in sham samples had completely changed, and only in very small specific areas, the original porosity of the scaffolds could be appreciated (Figure 8A). The great majority of the scaffold surface was completely covered by a thick and partially calcified tissue mantle that completely enveloped the exposed surface. In some areas, it could be confirmed an on-growth tissular neo-formation (Figure 8B).

At higher magnifications, it was possible to observe how the old pores were, in most cases, filled by a granular structure with an electron density similar but not identical to the scaffold, and that presumably corresponded to newly formed bone tissue (Figure 8C), as it was subsequently confirmed by genomic expression.

**Figure 9:**
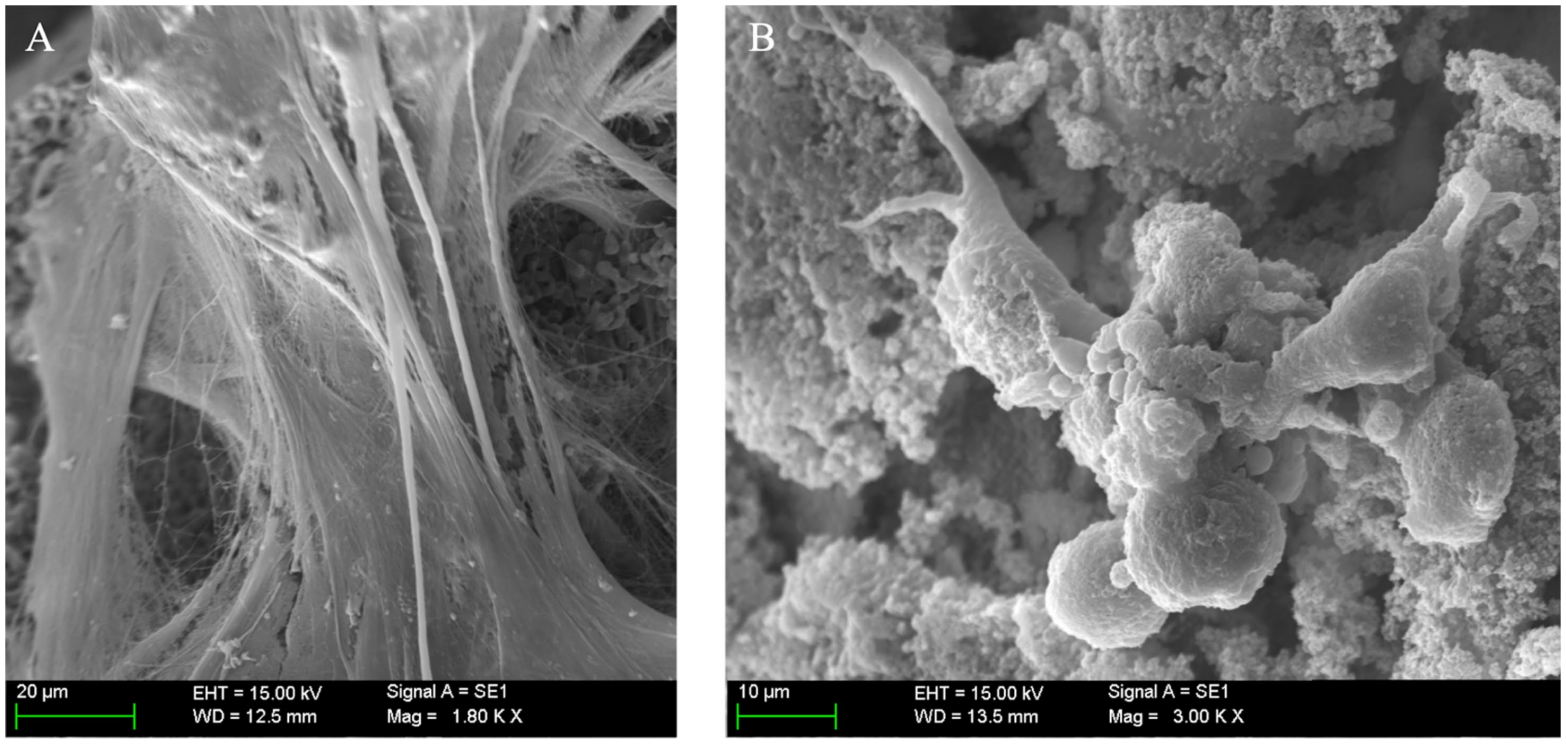
**(A)** Matrix deposition, mineralization, and osteoblast cell behavior in porous structures; **(B)** Scanning electron microscopy (SEM) at high magnification, clearly depicting mineralized extracellular matrix (ECM) with embedded mesenchymal stem cells (MSCs) covering a vast portion of the implant’s surface (scale bar: 20 µm).

Specialized cells (presumably osteoblasts) were seen congregating over seemingly covered pores (Figure 8D), while other cells embedded themselves in this dense ECM. These were considered functional osteoblasts responsible for the synthesis of bone tissue that filled the pores (Figure 8E) and, in more advanced phases, produced osteoid tissue with mature ECM (Figure 8F). Finally, at very high magnification, clustered nodular structures were also identified in certain areas, which were considered foci of calcium crystal precipitates and ECM deposition in densely populated areas (Figure 9).

#### 3.2.3. Genomic confirmation of MSC osteoblastic differentiation in 3D β-TCP scaffolds

Osteogenic differentiation was observed in all bio-active scaffolds under the conditions detailed for group S2, these samples displayed a baseline expression of multipotency-associated genes OCT4 and NANOG in their undifferentiated low-passage state at the time of seeding, nonetheless, after 21d osteogenic induction conditions in our scaffolds a shift was observed in the genomic expression towards a profile characteristic of osteogenic cells, evidenced by the upregulated expression of OC and OSX pre-osteoblast and osteoblast associated genes, and when compared with the expression of the canine housekeeping gene GAPDH. Although the quantification of expression for inter-sample comparisons was beyond the scope of this study, the mere confirmed presence of these genes along with intrinsic ECM-producing capabilities, noticeable morphology changes and loss of stem-related immunophenotype confirmed *in-vitro* osteoblastic activity.

## 4. Discussion

Bioceramics are a promising tool for bone regeneration, as they consistently provide evidence of osteoconductive and osteoinductive *in vitro* and *in vivo* properties. In this study, β-TCP foams performed as suitable *in-vitro* hosts for adipose-derived canine MSCs; moreover, cells did not only attach to the implants but also thrived, replicated, and differentiated into osteoblasts while promoting robust ECM and mineral deposition. Our host ceramic constructs with a mean macropore size of 232 μm performed similarly to human MSC studies, where pores (greater than 300 μm) were associated with increased vascularization of constructs and bone ingrowth, while pores smaller than 300 μm were thought to encourage osteochondral ossification (52, 53).

Even though the efficiency of static seeding and proliferation is reported to be inferior to that of bioreactors and fluidic systems (54, 55), it is demonstrated that in our conditions a considerable number of CD90-positive canine stem cells adhered to the ceramic foam’s surface and pores, and after only 72 h of a simple static culture. It is important to notice that the sufficient cell seeding density necessary to elicit a desired clinical outcome was beyond the scope of this study and it would have to be investigated in adequately designed clinical trials involving canine patients. Also, the presented digital seeding density estimation in 3D implants is a novel proposal in development, designed for veterinary sciences and potentially translatable to human regenerative medicine requiring standardization and refinement for validation purposes.

Nucleus DNA staining with DAPI blue although seldomly reported in previous canine MSC scaffold seeding research, proved convenient and useful because it allowed for the construction of a compound layered image that permitted cell counting and qualitative evaluation, while visually confirming the unequivocal expression of CD90 homogeneously throughout cell membranes in multipotent MSCs from S1 group. In contrast, expression of multipotency associated marker CD90 was lost and not detected in differentiating cells from S2 group, agreeably because its phenotype shifted to a more specialized profile, in accordance with observations in human MSCs where undifferentiated cell expression of CD90 was associated with enhanced osteogenic capacity when induced to differentiate. What is more, the detection of OSX and OC genes expression in our S2 group provided landmark evidence of the presence of these transcription factors in all processed samples, these being characteristically found in differentiated osteoblasts in canines (14) and associated with mineral formation, ECM deposition, and thus new bone formation.

Moreover, advanced evaluation of the samples by SEM allowed an accurate ultrastructural and topographical understanding of the surface of the scaffolds and the distribution of seeded cells. Further elucidation of microscopic changes in cells adhered and differentiating in tridimensional scaffolding may prove useful to understand the clinical reach of this association and to resolve emerging questions regarding cellular biological behavior (56). Overall, cells observed in confocal microscopy and SEM were considered congruent with previously published observations on morphology, migration and colonization of fracture sites during bone formation (52, 57).

Data derived from *in vitro* preclinical POC studies is intended to discover and establish aspects of the biological activity of medical products, and it is readily employed in the design of cytotoxicity and preclinical toxicology studies and clinical trials. In all cases, preliminary information on biocompatibility, proliferation and biological behavior, contributes greatly to defining reasonable risks to be assessed in product pre-clinical safety and efficacy studies (58). This work has several shortcomings, among them its small-sized sample, the lack of complimentary cellular viability determination methods for quantitative assessment of seeding efficiency, and objective comparisons with other 3D culture methods. Another topic to be addressed is the *in-vitro* implant pre-coating with PDL, which is an animal-free synthetic ECM yet not approved for human or animal consumption or use; this hurdle is itself an opportunity to further improve this prototype with various options of surface treatment. Options such as biocompatible polymer synthetic coatings (59), allogenic blood plasma derivatives such as platelet-rich fibrin or platelet lysates (60), and surface treatments such as homogenized collagen (61) provide an enhanced cellular attachment that could help with regulatory compliance, biocompatibility and safety.

Market and clinical feasibility require following a steep regulatory compliance path. Regardless of the implications of product registration, ethical manufacture of cell-containing implants implies also ethical obtention of primary tissues for isolation in carefully selected donors, thorough characterization of canine MSCs expanded in xeno-free cultures, and final product validation and depiction, so that patient owners and veterinarians are informed of what is the actual source, composition, and safety of the bioactive implant.

## 5. Conclusion

In our hands, foam-like scaffolds made with β-TCP bio-ceramics proved to be suitable carriers and hosts of canine adipose-derived MSCs, meaning that it’s possible to deliver multipotential cells to injury sites in a vessel structure that double acts as a cell guide, inducing cell invasion, attachment and specialization into bone healing osteoblasts. Parameters such as the loss of CD90 expression in cell membranes, biological and morphological changes, apparent ECM mineral deposition and expression of OC and OSX pre-osteoblast and osteoblast-associated genes confirmed successful *in situ* osteogenic differentiation in MSC populations from four different canines, suggesting a potential for reproducibility and replicability.

In the future veterinary clinical setting, these novel ortho biologic products may be of use in cases of bone healing impeachment such as delayed unions and non-unions, but also for pan-carpal and pan-tarsal arthrodesis as a fresh autologous bone graft volume expander or as a bioactive BGS. Although this research provides satisfactory *in-vitro* validation for the conceptualization and feasibility of a canine bio-active bone implant, further testing such as patient safety, large-scale reproducibility, and quality assessment are needed to advance in the long road toward clinical application and validation of osteointegration in veterinary pre-clinical models and patients.

## Data availability statement

The original contributions presented in the study are included in the article/supplementary material, further inquiries can be directed to the corresponding author.

## Author contributions

DH contribution involved all aspects of the project, including experimental design, development of protocols, standards of procedure, budgeting, execution of all experimental procedures, cellular cultures, cell sampling, staining, labeling, freezing for storage, and scaffold surface treatment. Also, image obtention, procurement, storage, and data interpretation. Finally, DH contributed to content writing on all sections of this paper, also in image processing, formatting, and referencing. JF contribution was the overall supervision, guidance, and experimental design counseling. Also, accessing departmental and internal funding, and acting as resource coordinator for laboratories and materials. Contributed to the main text body with image description and interpretation, results interpretation, content editing, coordinating reviews by external proofreading services, and supervising image obtention and editing. KR has contributed to protocol confection, data procurement, data analysis, and as an adviser on bone-compatible material configurations and pre-clinical research design. Also proofreading and image editing. M-PG contributed as an expert advisor in biomaterials. Responsible for the methodology, confection, production, and characterization of scaffolds. Other contributions include content writing on methods, editing, and proofreading. IL-T responsible for the methodology, confection, production, and characterization of scaffolds constructs. All authors contributed to the article and approved the submitted version.

## Conflict of interest

The authors declare that the research was conducted in the absence of any commercial or financial relationships that could be construed as a potential conflict of interest.

## Publisher’s note

All claims expressed in this article are solely those of the authors and do not necessarily represent those of their affiliated organizations, or those of the publisher, the editors and the reviewers. Any product that may be evaluated in this article, or claim that may be made by its manufacturer, is not guaranteed or endorsed by the publisher.
